# Bioinspired stability enhancement in deuterium-substituted organic–inorganic hybrid perovskite solar cells

**DOI:** 10.1093/pnasnexus/pgad160

**Published:** 2023-05-16

**Authors:** Jinhui Tong, Xun Li, Jianxin Wang, Haiying He, Tao Xu, Kai Zhu

**Affiliations:** Chemistry and Nanoscience Center, National Renewable Energy Laboratory, Golden, CO 80401, USA; Department of Chemistry and Biochemistry, Northern Illinois University, DeKalb, IL 60115, USA; Department of Chemistry and Biochemistry, Northern Illinois University, DeKalb, IL 60115, USA; Department of Physics and Astronomy, Valparaiso University, Valparaiso, IN 46383, USA; Department of Chemistry and Biochemistry, Northern Illinois University, DeKalb, IL 60115, USA; Chemistry and Nanoscience Center, National Renewable Energy Laboratory, Golden, CO 80401, USA

**Keywords:** deuterium, hybrid perovskite, stability, solar cells, degradation, kinetics

## Abstract

In hybrid perovskite solar cells (PSCs), the reaction of hydrogens (H) located in the amino group of the organic A-site cations with their neighboring halides plays a central role in degradation. Inspired by the retarded biological activities of cells in heavy water, we replaced the light H atom with its abundant, twice-as-heavy, nonradioactive isotope, deuterium (D) to hamper the motion of H. This D substitution retarded the formation kinetics of the detrimental H halides in Pb-based PSCs, as well as the H bond-mediated oxidation of Sn^2+^ in Sn–Pb-based narrow-bandgap PSCs, evidenced by accelerated stability studies. A computational study indicated that the zero point energy of D-based formamidinium (FA) is lower than that of pristine FA. In addition, the smaller increase in entropy in D-based FA than in pristine FA accounts for the increased formation free energy of the Sn^2+^ vacancies, which leads to the retarded oxidation kinetics of Sn^2+^. In this study, we show that substituting active H with D in organic cations is an effective way to enhance the stability of PSCs without sacrificing photovoltaic (PV) performance. This approach is also adaptable to other stabilizing methods.

**Significance Statement**

The slow motion of deuterium (D) in the amino group of A-site cations of hybrid perovskite impedes the detrimental formation of hydrogen halides and thus retards the degradation kinetics of hybrid perovskite. D substitution also improves the stability of narrow-bandgap perovskites by impeding the hydrogen bond-mediated Sn^2+^ oxidation to Sn^4+^, rendering a retrofit adaptable to all other existing stabilizing methods for hybrid perovskites and inspiring an alternative way to slow down a variety of materials degradation kinetics relevant to hydrogen mobility.

## Introduction

The certified champion power conversion efficiency (PCE) of perovskite solar cells (PSCs) is currently on par with or greater than that of silicon (Si)- and copper indium gallium selenide (CIGS)-based solar cells. In addition, solution processing makes PSCs a low-cost photovoltaic (PV) technology. However, there are still major challenges in achieving future widespread market adoption of PSCs, particularly their limited long-term stability. PSCs suffer from ∼5–10% PCE degradation over a few thousand hours under continuous operation (1–3). In contrast, Si-based solar cells exhibit a >20-year lifetime warranty with an average PCE degradation rate of only 0.5% per year.

The general chemical formula of metal halide perovskites is ABX_3_, where A is a monovalent mono, double, or triple cation—including organic CH_3_NH_3_^+^ (MA^+^) and CH(NH_2_)_2_^+^ (FA^+^) and/or inorganic Cs^+^, Rb^+^, and K^+^. The A-site cations of state-of-the-art Pb-based PSCs typically use organic cations with N–hydrogen (H) and C–N bonds. The rotating and stretching modes of these bonds have essential contributions to the high dielectric response and the high stability of excitons that retard the recombination rate through reorientation of organic cations (4–7). Explicitly, the rotating and stretching modes of the polar C–N and N–H bonds of the organic cations inside the inorganic PbX_6_^−^ cages deform and agitate, stabilizing the photoexcited inorganic framework through their electrostatic interactions with the inorganic lattice (4, 7). However, the polar N–H bond is the most vulnerable bond in hybrid perovskites under stress factors (e.g. potential, moisture, radiation, heat, and oxygen) (8–15). Polar N–H bonds split easily because the H atom is the lightest atom and can readily migrate to trigger a series of unwanted degradation reactions that lead to instability of PSCs, including the formation of HO_2_ with O_2_ (16–18) and/or H–I (19–21). The former proceeds to form Pb(OH)_2_ and PbO with Pb, and the latter disintegrates to H_2_ and I_2_, which can readily escape from the reaction center via defects in the film, further pushing the equilibrium toward degradation.

The stability issue becomes even more prominent in high-efficiency all-perovskite tandem solar cells because of the use of Sn–Pb mixed perovskite materials. Sn–Pb narrow-bandgap PSCs have achieved PCEs exceeding 20% (22). However, due to the strong inert pair effect in Sn^2+^ relative to Pb^2+^, the oxidation potential of Sn^2+^ → Sn^4+^ + 2*e*^−^ (+0.15 V) is much lower than that of Pb^2+^ → Pb^4+^ + 2*e*^−^ (+1.67 V). The unavoidable oxidation of Sn^2+^ to Sn^4+^ is a primary cause of the poor long-term stability of Sn-containing PSCs (23–25). The oxidation of Sn^2+^ to Sn^4+^ is relevant to the formation of H bonds between organic A-site cations and ingress H_2_O molecules and to the impact of A-site cation-induced polarization on the defect formation energy that regulates the emergence of Sn^2+^ from bulk to surface. Specifically, the organic cation in perovskites can adsorb H_2_O via the H bonds between the N (or H) from organic cations and the H (or O) from H_2_O (26). The stronger H bond of N^…^H can more tightly lock H_2_O with the A-site cation so as to slow down H_2_O-mediated oxidation of Sn^2+^ to Sn^4+^; the weaker H bond of H^…^O, however, allows relatively faster H_2_O-mediated migration of O_2_ to the deep lattice, thus faster oxidation of Sn^2+^ to Sn^4+^ (26–28). In parallel, the oxidation of a surface Sn^2+^ by O_2_ generates a Sn^2+^ vacancy due to the migration of the resulting SnO_2_ (29, 30). As a result, the inward polarization (due to vibration and/or rotation of polar bonds such as N–H) of A-site cations leads to a downhill slope of defect formation energy for Sn^2+^ vacancies from surface to subsurface layers, causing Sn^2+^ to emerge from subsurface layers to the surface (29–34). It is thus evident that the unwanted motion of H is a notable root cause of the instability in PSCs containing organic A-site cations and Sn^2+^.

Inspired by the emerging longevity aging study in biological science, particularly the slow growth of yeast cells in heavy water (35), we herein report a nuclear approach to retard any H-dependent degradation reactions in both Pb-based and Sn–Pb-based PSCs by replacing H with its heavier but nonradioactive and resourceful isotope deuterium (D). Because D is twice as heavy as H, the kinetics of all the degradation mechanisms related to the motion of H can be substantively slowed down, leading to a marked enhancement in device stability in accelerated aging tests. In particular, the replacement of H by D can notably attenuate the frequency of the H bonds by a factor of (2)^1/2^ and lower the zero point vibrational energy in the H bonds (27). The smaller dipole moment and slightly shorter bond length of D–N compared to H–N (32, 33) retard the polarization of the neighboring inorganic framework—namely, the emergence of Sn^2+^ from the subsurface to the surface by A-site organic cations—so as to reduce the oxidation of Sn-containing perovskites. Note that the replacement of N–H with N–D can be conveniently achieved by soaking and recrystallizing the organic precursors [e.g. methylammonium iodide (MAI) and formamidinium iodide (FAI) in D_2_O]. Ocean water provides a source of D that is more than sufficient to supply the needs of any future PSC-based PV applications (see technoecono analysis of D-based perovskite in Note S1).

## Results and discussion

We first study the inertness of D-substituted FA and MA using the Tyndall effect, which is a direct, visual method for distinguishing the stabilities of H-based organic cations from those of D-based organic cations. We prepared two perovskite precursory solutions for comparison, including an H-based solution containing MAI (or CH_3_NH_3_I), butylamine [BA or CH_3_(CH_2_)_3_NH_2_], and PbI_2_; and a D-based precursory solution containing deuterated MAI (D–MAI or CD_3_ND_3_I), BA, and PbI_2_. Both solutions have a molar ratio of 1:1:1 for the three ingredients at a concentration of 200 mg/mL with reference to PbI_2_. Figure. S1 shows that initially, both solutions appeared homogeneous and no Tyndall effect was observed. However, after the solutions were aged on a hot plate at 55°C for 8 days, precipitates formed in the D-MAPbI_3_ precursor solution, clearly visualized by the Tyndall effect. The Tyndall effect was observed only in the precursor solution containing CD_3_ND_3_PbI_3_. The reaction mechanism is outlined below, with the downward arrow used to indicate the precipitation.

(Reaction 1)


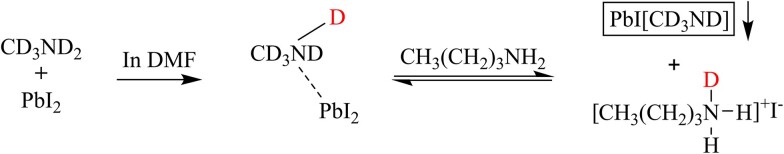


(Reaction 2)


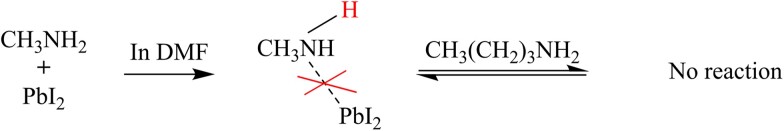
In reaction 1, due to the low acidity of the D-based MA (owing to the retarded motion of D), the D-based MA behaves as a base and forms a complex with PbI_2_ through a Lewis acid-base reaction (the Pb^2+^ cation is the Lewis acid, and the lone pair of electrons on the N of the D-based MA is the Lewis base). This reaction outpaces the reaction between the Lewis acid Pb^2+^ and the Lewis base dimethylformamide (DMF) molecules. The resulting complex (CD_3_ND_2_^…^PbI_2_) is acidic enough (N–D is activated) to be deprotonated by a strong base of BA (donor number = 42 kcal/mol). However, it cannot be deprotonated by DMF, because DMF (donor number = 26 kcal/mol) is a weaker base than BA. Finally, a precipitate of D–PbI–methylamide iodide (the conjugated base of the CD_3_ND_2_^…^PbI_2_ complex) and a salt of D-butylammonium iodide (the conjugate acid of BA) is generated (36). In the case of reaction 2, due to the faster motion of H than D and possibly the lower dissociation energy of N–H than N–D (37), the H-based MA exhibits higher acidity than D-based MA or DMF molecules—in other words, H-based MA is a weaker base than D-based MA or DMF. Thus, the Lewis acid Pb^2+^ preferentially reacts with the lone pair of electrons on O of the DMF molecules (a Lewis base), instead of reacting with the weaker Lewis base of the H-based MA. As a result, there is no subsequent intermediate complex formed because of the complete solvation of Pb^2+^ by DMF molecules.

**Fig. 1.**
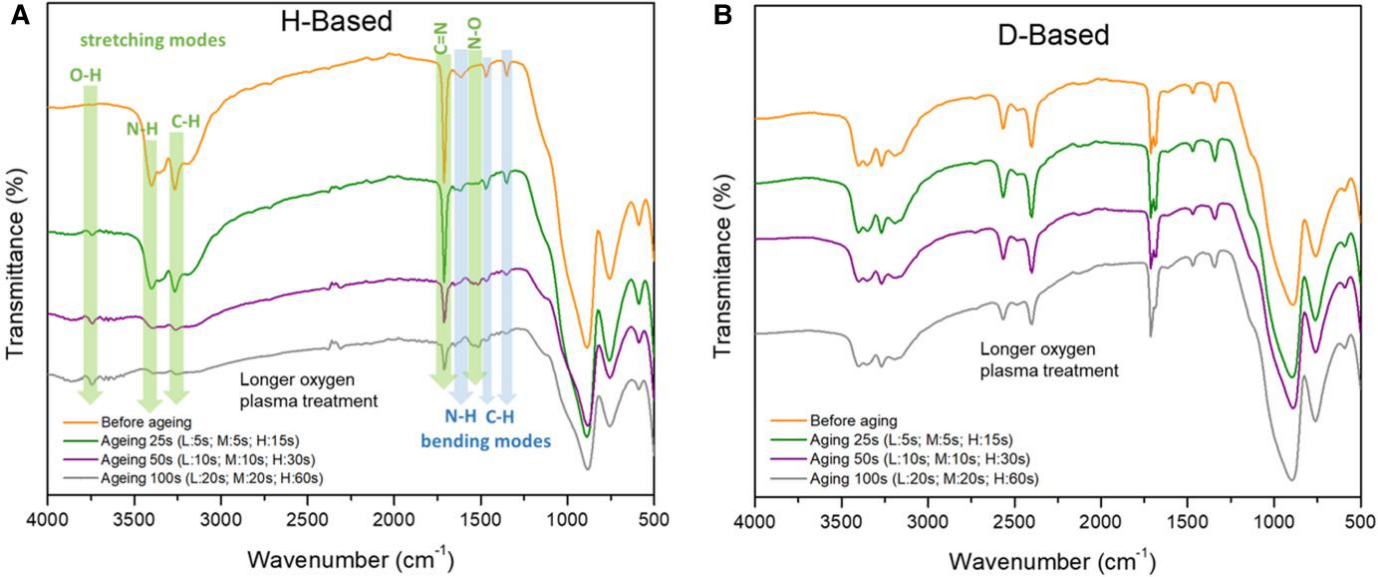
A) FTIR of H-based perovskite films (composition: (H-FASnI_3_)_0.6_(H-MAPbI_3_)_0.4_) before and after oxygen plasma treatment for different aging durations. Three levels of oxygen plasma intensity were used (L, low energy; M, medium energy; H, high energy). B) FTIR of D-based perovskite films (composition: (D-FASnI_3_)_0.6_(D-MAPbI_3_)_0.4_) before and after oxygen plasma treatment using the same aging conditions as in the H-based perovskite film.

We further assessed the antioxidation property between H-based perovskite and D-based perovskite films by exposing the perovskite films to highly energetic oxygen plasma for accelerated oxidation. Two different perovskite compositions were used: (CH(NH_2_)_2_SnI_3_)_0.6_(CH_3_NH_3_PbI_3_)_0.4_ (denoted as “H-based”) and (CH(ND_2_)_2_SnI_3_)_0.6_(CD_3_ND_3_PbI_3_)_0.4_ (denoted as “D-based”). Fourier transform infrared spectroscopy (FTIR) was used to characterize the change of functional groups in the perovskite thin films before and after oxygen plasma treatment. With increasing oxygen plasma treatment time, FTIR of the H-based perovskite film (Fig. 1A) showed attenuated intensities for peaks at ν ≈ 3,402 cm^−1^ (N–H stretching), ν ≈ 3,271 cm^−1^ (C–H stretching), ν ≈ 1,712 cm^−1^ (C = N stretching, characteristic to FA), ν ≈ 1,612 cm^−1^ (N–H bending), and ν ≈ 1,470/1,350 cm^−1^ (C–H bending). These results indicate the oxidation of these functional groups, namely, the insertion of oxygen atoms, which is further evidenced by the corresponding growth of two new peaks at ν ≈ 3,749 cm^−1^ (free O–H stretching) and ν ≈ 1,519 cm^−1^ (N–O stretching) with increasing oxygen plasma treatment time. After 100 s of oxygen plasma treatment, only the O–H stretching, N–O stretching, and part of C = N stretching bonds could be detected. In a remarkable contrast, almost no changes were observed in the FTIR of the D-based samples, as shown in Fig. 1B, even after 100 s of oxygen plasma treatment. This comparison indicates that the N–D or C–D bonds are more inert than N–H or C–H bonds, due to the comparative heaviness of D, which impedes its motion and therefore its ability to accommodate the oxygen insertion.

**Fig. 2.**
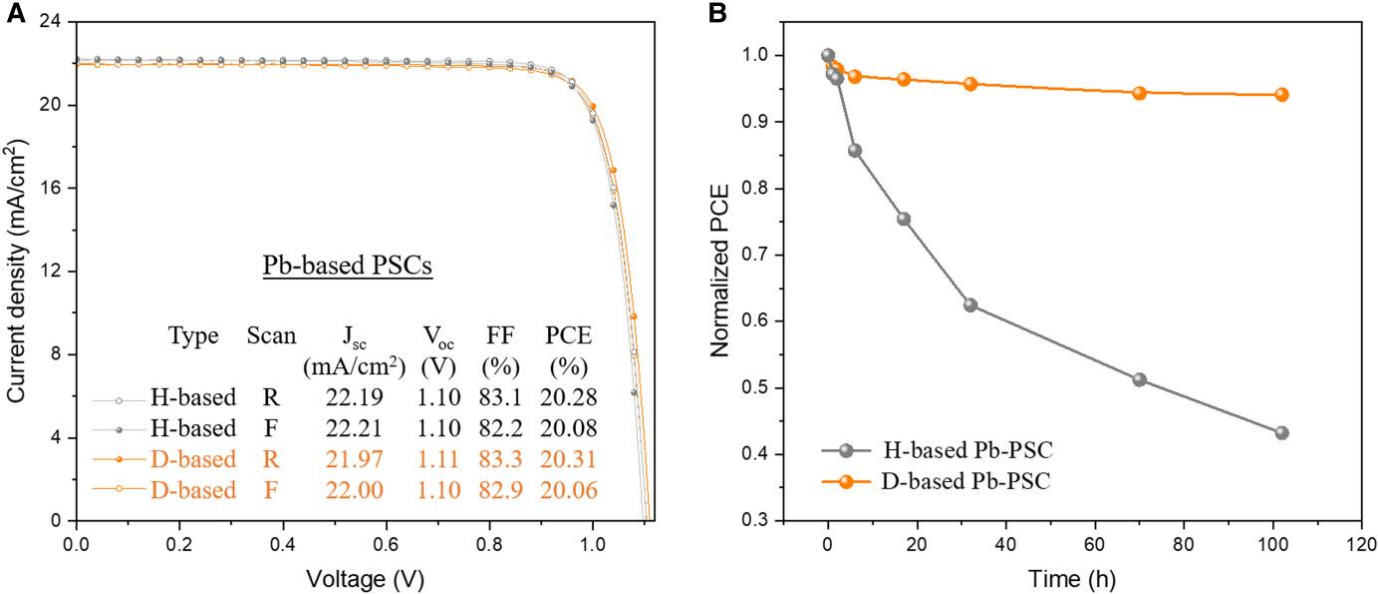
A) J-V characteristics with reverse (R) and forward (F) scans for H-based and D-based Cs_0.05_MA_0.15_FA_0.8_PbI_2.55_Br_0.45_ PSCs; B) thermal stability evaluation of the corresponding unencapsulated devices at 85°C in dry air.

To experimentally evaluate the effect of D-substituted organic A-site cations on the PV performance of PSCs, we first studied pure Pb-based perovskites with the composition of Cs_0.05_MA_0.15_FA_0.8_PbI_2.55_Br_0.45_ using a standard p-i-n configuration [indium tin oxide (ITO)/poly(triaryl amine) (PTAA)/perovskite/C_60_/bathocuproine (BCP)/Ag]. Figure 2A shows the comparable current density–voltage (J-V) curves from the H-based devices (both MA and FA are pristine H-based) and the D-based devices (both MA and FA are D-based). Note that neither type of device was encapsulated. Both H-based and D-based devices exhibited nearly the same J-V behaviors, with no observable hysteresis, indicating that deuteriation does not affect the PV performance of the devices. Figure 2B shows the device stability after thermal aging at 85°C for 150 h in dry air. Strikingly, the D-based devices retain most of their initial PCE, whereas the pristine H-based devices exhibit <40% <of their initial PCE.

To gain mechanistic insight into the enhanced thermal stability of D-based devices, we further calculated the vibrational frequencies of the N–H and N–D bonds in the H-based and D-based FA (see Table S1 and Fig. S2). The calculated N–H frequencies are in the range of 3,415–3,537 cm^−1^, in good agreement with the experiment. In contrast, the N–D frequencies are a factor of just 0.723–0.742 of the N–H ones, indicating that N–D vibration is drastically retarded. Therefore, the reaction kinetics involving the motion of H can be largely slowed down by replacing H with D, in good agreement with our experimental study. The calculated zero point energy (ZPE) of D-based FA is lowered by 0.36 eV (i.e. 4,123 K) relative to that of H-based FA. Because the electronic properties do not change with the substitution of H by D, the enhanced thermal stability of D-based PSCs can be logically attributed to the slow motion of D, which retards the kinetics of the degradation reactions.

Sn doping of perovskites enables promising narrow-bandgap PSCs (denoted Sn–Pb PSCs) for enhanced current density. To study the impact of D-substituted organic A-site cations on Sn-doped perovskites, we chose the MA-free perovskite composition of Cs_0.25_FA_0.75_Sn_0.5_Pb_0.5_I_3_ to avoid complications from the deleterious effect of MA^+^ volatility. Figure S3 shows the X-ray diffraction (XRD) patterns of Cs_0.25_FA_0.75_Sn_0.5_Pb_0.5_I_3_ using H–FAI (H-based) and D–FAI (D-based) as the organic A-site cation, respectively. The XRD patterns of both the H-based film and the D-based film show identical features in terms of peak position, peak intensity, and peak width, indicating that D substitution does not change film lattice structure and crystallinity. We also conducted scanning electron microscopy (SEM) to compare the film morphology, as shown in Fig. S4. This figure shows that both films exhibit similar representative grain size and film morphology. Thus, D substitution does not affect film morphology either.

**Fig. 3.**
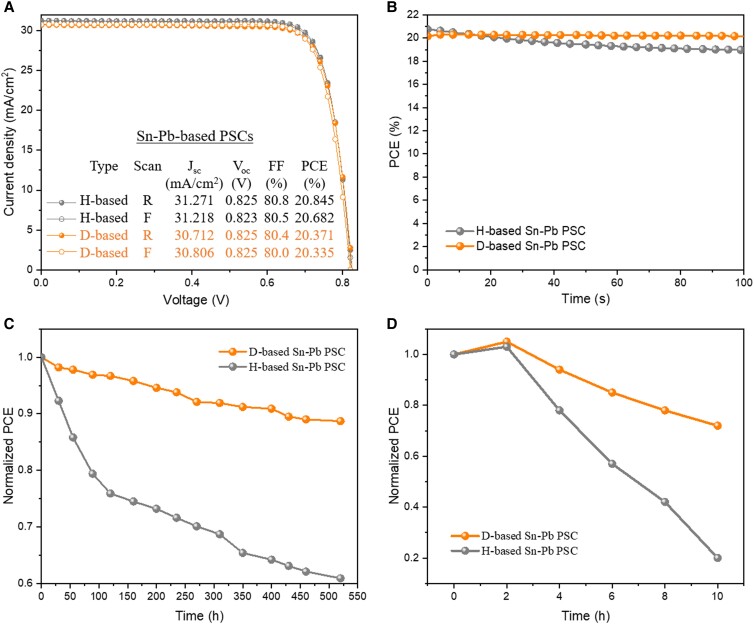
PV performance comparison of H–FAI- and D–FAI-based Cs_0.25_FA_0.75_Sn_0.5_Pb_0.5_I_3_ narrow-bandgap PSCs (all unencapsulated). A) Comparison of J-V curves. B) Comparison of SPO. C) Comparison of operational stability at RH = ∼50% and temperature of ∼50°C under continuous light illumination in air. D) Comparison of ambient stability of the Pd–Sn narrow-bandgap PCSs under 82% RH. Devices were not encapsulated.

Figure 3A compares the J-V curves of the narrow-bandgap PSCs based on Cs_0.25_FA_0.75_Sn_0.5_Pb_0.5_I_3_ with H–FAI and D–FAI as the organic A-site cations. Note that these narrow-bandgap PSCs were made under the same conditions and were not encapsulated. When measured under a reverse voltage scan, the narrow-bandgap PSC made with H–FAI exhibited a PCE of 20.845% with a short-circuit current density (*J*_sc_) of 31.271 mA/cm^2^, an open-circuit voltage (*V*_oc_) of 0.825 V, and a fill factor (FF) of 0.808. When measured under a forward voltage scan, the H–FAI-based narrow-bandgap PSC showed a PCE of 20.682% with a *J*_sc_ of 31.218 mA/cm^2^, a *V*_oc_ of 0.823 V, and an FF of 0.805. D-based devices show similar J-V performance to that of H-based devices, exhibiting a PCE of 20.371% for reverse scan and 20.335% for forward scan. Figure 3B compares the stabilized power output (SPO) efficiencies. H-based devices have an SPO efficiency of 20.750%, very close to the SPO efficiency of 20.158% for D-based devices. It is clear that both H-based and D-based narrow-bandgap PSCs show similar PV performance, indicating that the substitution of D for H does not affect the normal PV performance.

We further conducted an accelerated evaluation on the operational stability of the encapsulated narrow-bandgap PSCs in air with a relative humidity (RH) of ∼50% and a temperature of ∼50°C under continuous light illumination in air. Both H-based and D-based narrow-bandgap PSCs were biased near the maximum power point during illumination. As can be seen in Fig. 3C, the H-based Sn–Pb PSC showed about 29.3% efficiency loss after 550 h of illumination, whereas the D-based Sn–Pb PSC showed markedly enhanced stability, with only 11.4% degradation. We further tested the stability of unencapsulated H-based and D-based Sn–Pb narrow-bandgap PSCs in air (∼80% RH). As shown in Fig. 3D, the D-based Sn–Pb PSC showed notably better atmospheric stability than the H-based Sn–Pb PSC. The D-based Sn–Pb PSC retained 82% of its initial efficiency after 8 h of storage in humid air, whereas the H–FAI-based device retained only of 71% of its initial efficiency during the same period.

We also conducted computational modeling and simulation based on density functional theory (DFT) to provide a fundamental understanding of the remarkably enhanced antioxidation of Sn^2+^ to Sn^4+^ found in the D-based narrow-bandgap PSCs under humid conditions in open air. First, we constructed an FA^…^H_2_O complex model in order to investigate the impact of deuteration of the N–H bond in FA on the diffusion of H_2_O molecules into the bulk. In the equilibrium state, an H bond is formed as N–H^…^O (Fig. S5), which results in an H_2_O binding energy of 0.65 eV and an elongation of the N–H bond from 1.01 to 1.03 Å. Consequently, the vibrational frequency of the N–H stretching mode is reduced by a factor of ∼0.9 (Table S2 and Fig. S6). Substituting D for H further reduced the vibrational frequencies by a factor of ∼0.7, which in turn, slowed down the diffusion of water molecules.

**Fig. 4.**
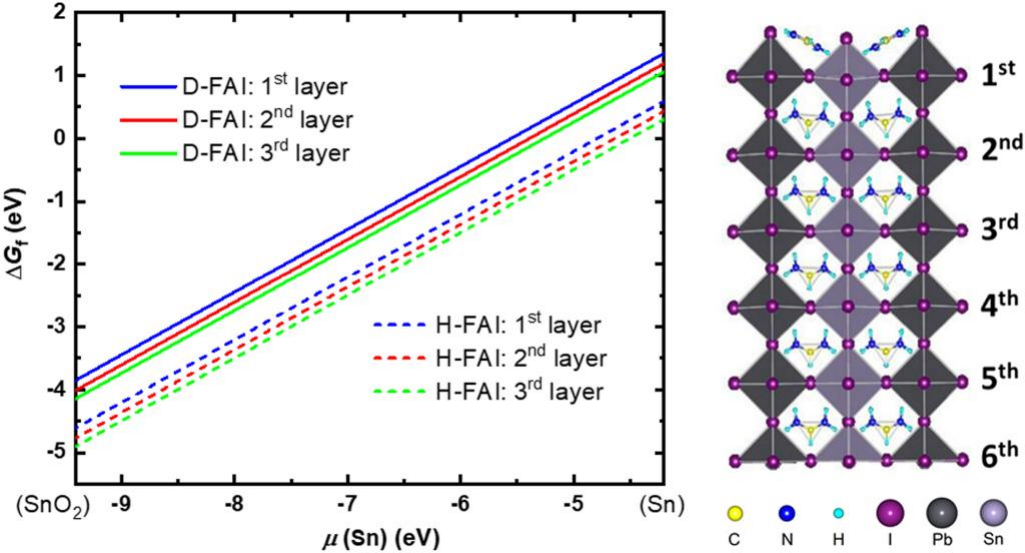
Sn^2+^ vacancy ($V_{\mathrm{Sn}}^{2-}$) formation free energies as a function of the Sn chemical potential *μ*(Sn) for vacancies located at different layers beneath the top surface layer of a model system of the FASn_0.5_Pb_0.5_I_3_ (001) surface.

The oxidation of Sn^2+^ into Sn^4+^ (i.e. SnO_2_) is most likely to start from the surface of the perovskite as it is exposed to water and oxygen in humid air. Earlier studies have shown that, regardless of the A-site cation in a hybrid ASnX_3_ perovskite, the reaction between Sn^2+^ and adsorbed O_2_ is energetically favorable, which leads to the formation of SnO_2_ and Sn^2+^ vacancies ($V_{\mathrm{Sn}}^{2-}$) at the outermost surface (30). The Sn^2+^ ions underneath may migrate to fill in the Sn^2+^ vacancies and form vacancies below. As this process continues, more Sn^2+^ ions propagate to the surface and get oxidized to Sn^4+^, while the Sn^2+^ vacancies move down into the bulk. Consequently, ASnX_3_ degrades to A_2_SnX_6_, destroying the PSC performance. Additionally, the study of MASnBr_3_ has demonstrated that the polarization induced by MA ions at the A-site with a specific orientation can significantly expedite the migration of $V_{\mathrm{Sn}}^{2-}$ from the surface to the bulk region compared to the nonpolar Cs ions (30). We employed a model system of the FASn_0.5_Pb_0.5_I_3_ (001) surface to investigate the dependence of the defect formation energy of $V_{\mathrm{Sn}}^{2-}$ on its location, the Sn chemical potential *μ*(Sn), and the deuteration effect. To study the dependence of the vacancy formation energy on the location of the vacancy underneath the surface, we generated the Sn vacancy in the first, second, and third layers of the (001) surface. It is interesting to note that the orientation of FA cations tilted near the Sn vacancy (Fig. S7). To reveal the deuteration effect by substituting D for H in the FA molecule, we calculated the vibrational frequencies of the system, thereby obtaining the free energy corrections (at *T* = 298.15 K) to the formation energy of $V_{\mathrm{Sn}}^{2-}$ (see Table S3). The calculated formation free energies (Δ*G*_f_) of $V_{\mathrm{Sn}}^{2-}$ at different depths (layers) beneath the top surface layer are plotted as a function of *μ*(Sn) in Fig. 4. The canted lines indicate the change of formation energies as *μ*(Sn) increases from the equivalent chemical potential of Sn in the SnO_2_ bulk (O_2_-rich) to that of a Sn-rich environment. Note that the positive formation energy indicates that the formation of a vacancy requires energy, whereas the negative formation energy at lower *μ*(Sn) suggests a spontaneous process.

These results show a similar trend as the previous study (30), where at a certain *μ*(Sn), Δ*G*_f_ decreases with the increase in depth of the Sn vacancy location, along the same direction as the polarization caused by the FA cations. However, the difference in the value of Δ*G*_f_ is smaller than in the case of MASnBr_3_. This is likely due to the fact that the FA cation has a much smaller intrinsic dipole moment (0.081 Debye) than the MA cation (0.845 Debye), in agreement with the observed better stability of FA. In addition, the deuteration of FA increases the formation free energy of $V_{\mathrm{Sn}}^{2-}$ by 0.76 eV, largely due to the smaller increase in entropy in the defective system with $V_{\mathrm{Sn}}^{2-}$ than in the pristine surface without vacancies (see Table S3). These results reveal that the adoption of a D-substituted organic component reduces the degree of oxidation of the Sn^2+^ cations.

In summary, the replacement of H—particularly the active H in the amino group—with D is an effective approach to reducing the vibration frequency of the N–D bond that is accountable for the degradation kinetics of perovskites under stress factors such as heat and light. The deuteration of organic cations also reduced the entropy change in D–FA. This helped increase the formation free energy of the Sn^2+^ vacancies, impeding the emergence of Sn^2+^ to the surface layer where oxidation occurs. Thus, it is evident that substitution of active H with D in organic cations is a standalone kinetic method to enhance the stability of PSCs, as well as a conveniently adaptable retrofit to any other stabilizing methods that do not require changing procedures or compositional formula.

## Materials and methods

### Synthesis of D-replaced FAI and MAI

#### Synthesis of deuterated CH(ND_2_)_2_I (D–FAI)

CH(NH_2_)_2_I (FAI, Greatcell Solar Materials Pty Ltd.) was dissolved in excessive D oxide (Sigma-Aldrich, 99.9 atom %) at a molar ratio of 1:40 under stirring, followed by heating the solution to 90°C for 2 h under argon (Ar). The liquid was then evaporated at 70°C under a flow of Ar to dry off. This process was repeated three times to ensure complete substitution of ammonium H by D to yield the high purity of D–FAI. Finally, the white solid was collected and moved to the vacuum oven at 60°C overnight for further drying. In principle, most of the D_2_O can be recollected by reflux for future scaled-up synthesis.

#### Synthesis of deuterated CD_3_ND_3_I (D–MAI)

First, CD_3_NH_2_ gas (Sigma-Aldrich, 99.9 atom %) was slowly reacted with an equimolar amount of hydroiodic acid (HI) solution (Sigma-Aldrich, 57 wt% in water) in a round-bottom flask soaked in an ice bath. Then, the solvent of this solution was dried off by rotary evaporation at 60°C. The collected solid of CD_3_NH_3_I with HI residual was further washed by anhydrous ethyl ether (Fisher Chemical) under vacuum filtration, followed by drying the white solid in the vacuum oven at 60°C overnight. Next, the collected CD_3_NH_3_I was dissolved in excessive D oxide at a molar ratio of 1:40 with stirring, followed by heating the solution to 90°C for 2 h under Ar and then evaporating the solution at 70°C under flowing Ar to dry off the solvent. This process was repeated three times to ensure complete substitution of N–H by N–D to yield the high purity of D–MAI. Finally, the white solid was collected and transferred to the vacuum oven at 60°C overnight for further drying. All samples were characterized by nuclear magnetic resonance (NMR) spectroscopy, performed using a Bruker Spectrospin 500-MHz NMR spectrometer. Figures S8–S10 show the NMR results of CH(NH_2_)_2_I, CH(ND_2_)_2_I, and the solvent [deuterated dimethyl sulfoxide (DMSO)] used in the NMR study, respectively. NMR results for CH_3_NH_3_I and CD_3_ND_3_I were reported in our previous work (38).

### Oxygen plasma oxidation of perovskite films

The accelerated oxidation of different perovskite films was conducted using a plasma cleaner (PDC-32G, Harrick Plasma). This plasma cleaner includes three gears: low energy, medium energy, and high energy.

### Preparation of PSC precursor

All the perovskite solution and film depositions were conducted in a N_2_ glovebox with O_2_ level < 1 ppm and H_2_O < 1 ppm. For the H-based Pb–Sn mixed narrow-bandgap Cs_0.25_FA_0.75_Sn_0.5_Pb_0.5_I_3_ PSC precursor, the solution consisted of 258-mg H–FAI (pure hydrogenated FAI, Greatcell, Australia), 461-mg PbI_2_ (anhydrous, Sigma-Aldrich, USA), 130-mg CsI (anhydrous, Sigma-Aldrich, USA), 16-mg SnF_2_ (Sigma-Aldrich, USA), and 338.7-mg SnI_2_ (anhydrous, bead, Sigma-Aldrich, USA) in 800-mL DMF (anhydrous, Sigma-Aldrich, USA) and 200-mL DMSO (anhydrous, Sigma-Aldrich). To prepare the corresponding D-based Pb–Sn mixed narrow-bandgap Cs_0.25_FA_0.75_Sn_0.5_Pb_0.5_I_3_ perovskites, we used 264-mg D–FAI instead of 258-mg H–FAI and 264-mg D–MA instead of 258-mg H–MA. Similar method was used to prepare the D-based Cs_0.05_FA_0.8_MA_0.15_PbI_2.55_Br_0.45_. In the case of D-based Cs_0.05_FA_0.8_MA_0.15_PbI_2.55_Br_0.45_, equal moles of D–FAI and D–MA were used instead of H–FAI or H–MAI.

### PSC device fabrication

For Pb–Sn mixed narrow-bandgap PSCs, the prepatterned ITO substrates were sequentially cleaned by ultrasonication in acetone and isopropanol three times. The ITO/glass substrates were then dried with an N_2_ gun and were UV-ozone treated for 15 min. The poly(3,4-ethylenedioxythiophene):polystyrene sulfonate (PEDOT:PSS) (Clevios P VP Al 4083, filtered through a 0.45-mm nylon filter) were spin-coated onto ITO substrates at 3,000 rpm for 30 s and annealed on a hot plate at 150°C for 30 min in air. After that, the substrates were transferred to a nitrogen glovebox (O_2_ level < 1 ppm, H_2_O < 1 ppm) to prepare the narrow-bandgap perovskite layer. The Cs_0.25_FA_0.75_Sn_0.5_Pb_0.5_I_3_ perovskite films were deposited by spin-coating the solution as described above at 5,000 rpm for 40 s. An N_2_ stream was blown over the spinning substrate for 20 s during the spinning procedure to assist with the formation of the perovskite film. Films were then annealed at 120°C for 10 min. Finally, C60 (30 nm), BCP (6 nm), and Ag (100 nm) were sequentially deposited by a thermal evaporator to complete the PSCs.

For Cs_0.05_FA_0.8_MA_0.15_PbI_2.55_Br_0.45_ PSCs, the prepatterned ITO substrates (15 Ω/sq) were sequentially ultrasonic cleaned using acetone and 2-propanol. The ITO substrates were then transferred into the nitrogen-filled glovebox. Then, 2 mg/ml PTAA solution was spin-coated onto the ITO substrates at 5,000 rpm for 30 s and annealed at 100°C for 10 min. The Cs_0.05_FA_0.8_MA_0.15_PbI_2.55_Br_0.45_ perovskite precursor was spin-coated onto the PTAA/ITO substrate at 5,000 rpm for 30 s. After 10 s of spin-coating, 350-*µ*L diethyl ether (DEE) was dropped onto the substrate. The resulting perovskite films were then annealed at 100°C for 10 min. After the deposition of the perovskite film, C60 (30 nm), BCP (6 nm), and Ag (100 nm) were sequentially deposited by thermal evaporation.

### Characterization

Devices were tested using a Newport Oriel Sol 3A solar simulator with a xenon lamp in a nitrogen-filled glovebox. The intensity of the solar simulator was calibrated to 100 mW/cm^2^ AM 1.5G. The light current J-V characteristic was taken with a step size of 10–30 mV and a step delay of 10 ms, unless otherwise stated. The device area was 0.1 cm^2^, and the device was masked with a metal aperture to define an active area of 0.058 cm^2^.

### Computational method

#### Cluster calculations

Electronic structure calculations based on DFT were performed with the B3LYP functional form (39, 40) together with the 6-311 + G(3df,2p) basis sets using the program package Gaussian 09 (41). Contributions from vdW dispersion forces were included in the form of the Grimme-D2 terms in the calculations (42). The geometry optimization was done without applying any constraints. The convergence criteria for maximum force, RMS force, maximum displacement, and RMS displacement were set as 0.023 eV/Å, 0.015 eV/Å, 9.5 × 10^−4^ Å, and 6.4 × 10^−4^ Å, respectively. Vibrational frequencies were scaled by a factor of 0.9670 (43).

The electronic binding energy, *E*_b_, of the H_2_O molecule with an FA ionic compound is computed as


(1) $$E_{b}=(E_{\mathrm{FA}}+E_{H2O})-E_{\mathrm{FA}-H2O},$$

where *E*_FA-H2O_, *E*_FA_, and *E*_H2O_ are the total energies of the FA^…^H_2_O complex, the FA ionic compound, and the H_2_O molecule, respectively. The more positive the value of *E*_b_, the stronger the interaction.

#### Periodic (bulk and surface) calculations

We also carried out FASn_0.5_Pb_0.5_I_3_ bulk and surface calculations in the framework of DFT by applying periodic boundary conditions using the electronic structure code VASP (44–47). The PBE exchange-correlation functional (48) was adopted together with the van der Waals (vdW) interactions described via a pairwise force field using the DFT-D3 method of Grimme (49) with Becke–Johnson damping (50), where the improved dispersion coefficients *C*_6*ij*_ are local geometry dependent. The projector-augmented wave (PAW) method and plane wave basis sets were used with energy cutoffs of 520 eV for full-cell geometry optimization and 400 eV for geometry optimization with fixed cell parameters. The total energy was converged to 10^−5^ eV for each electronic step, and the force on each atom was converged below 0.03 eV/Å.

The FASn_0.5_Pb_0.5_I_3_ (001) surface was adopted to investigate the defect formation energies of Sn^2+^ vacancies $V_{\mathrm{Sn}}^{2-}$. A six-bilayer 2 × 2 slab was constructed with a thickness of ∼35 Å and a vacuum of 15 Å to ensure negligible interaction of the slab with its neighboring images. The internal coordinates of atoms were optimized while keeping the bottom two layers fixed to the bulk positions. The Brillouin zone was sampled using a 3 × 3 × 1 Monkhorst–Pack grid for integration in the reciprocal space for the pristine and defective systems. Note that for the system with $V_{\mathrm{Sn}}^{2-}$, two excess electrons were introduced into the supercell and charge was balanced by an uniform positive background.

The formation free energy of $\;V_{\mathrm{Sn}}^{2-}$ can be calculated as


(2) $$\Delta G_{f}=\mu (\mathrm{defect})+\mu (\mathrm{Sn})-\mu (\mathrm{pristine})-2\mu (e^{-}),$$

where *μ*(defect) and *μ*(pristine) are the Gibbs free energies of defective and pristine FASn_0.5_Pb_0.5_I_3_ (001) surfaces, respectively. These values are obtained by the computed electronic total energy *E* and the free energy correction Δ*G*_corr_, based on the frequency calculations.


(3) $$\mu (\mathrm{defect})=E(\mathrm{defect})+\Delta G_{\mathrm{corr}}(\mathrm{defect}),$$

(4) $$\mu (\mathrm{pristine})=E(\mathrm{pristine})+\Delta G_{\mathrm{corr}}(\mathrm{pristine}).$$

Here, *μ*(Sn) is the chemical potential of Sn, which is chosen to vary from the cohesive energy per atom of the Sn bulk to the chemical potential of Sn in the SnO_2_ bulk, and *μ*(e^−^) is the energy of an electron, which is set to the Fermi energy of the pristine surface.

#### Additional computational details and results

For better comparison with the experimental results (H-based and D-based Cs_0.25_FA_0.75_Sn_0.5_Pb_0.5_I_3_), we chose a model system, FASn_0.5_Pb_0.5_I_3_, that captures the major characteristics of composition and structure and also makes the computational task affordable. The FASn_0.5_Pb_0.5_I_3_ crystal is based on the crystalline structures of FAPbI_3_ (51), with half of the Pb ions replaced by Sn. The trigonal planar FA cations [HC(NH_2_)_2_]^+^ are set to lie in the central mirror plane of the unit cell with the C–H bond pointing into a cube face, whereas the −NH_2_ groups form a H bond with the I atoms. The preference of Sn ordering at the B-site is investigated by constructing a 2 × 2 × 2 supercell and sampling three different Sn ordering orientations along the three principal vectors in the perovskite conventional cell (Fig. S11). The total electronic energy only differs on the order of 0.0002 eV per formula unit (FASn_0.5_Pb_0.5_I_3_), suggesting that it is not sensitive to the ordering of substitution of Sn in the bulk. We chose the lowest energy configuration for the surface calculations.

Energetics were considered for four surface terminations: (001) surface with FAI termination, (001) surface with PbI_2_ termination, (00 $\bar{1}$) surface with FAI termination, and (00 $\bar{1}$) surface with PbI_2_ termination (see Fig. S12). The (001) surface has the −NH_2_ group (negatively charged) pointing toward the surface, whereas the −CH group (positively charged) points away from the surface. Thus, the intrinsic dipole moment of the FA ion points from the surface to the bulk region for the (001) surface and in the opposite direction for the (00$\bar{1}$) surface. The calculated total energy shows that the (001) surface with FAI termination has the lowest energy. Therefore, we chose it for the calculation of formation energies of Sn vacancies.

The Sn vacancy is formed by removing an Sn atom from the pristine surface. The Sn vacancy can carry different charge states—0, −1, and −2—with the −2 charge state ($V_{\mathrm{Sn}}^{2-}$) being the lowest in formation energy (*30*). To study the dependence of the vacancy formation energy on the location of the vacancy underneath the surface, we generated the Sn vacancy in the first, second, and third layers of the (001) surface. It is interesting to note that the orientation of FA cations tilted near the Sn vacancy. To reveal the deuteration effect by substituting D for H in the FA molecule, we calculated the vibrational frequencies of the system, thereby obtaining the free energy corrections (at *T* = 298.15 K) to the formation energy of $V_{\mathrm{Sn}}^{2-}$ (see Table S3). The positions of the Pb, Sn, and I atoms are constrained in these frequency calculations.

## Supplementary Material

pgad160_Supplementary_Data

## Data Availability

All data are included in the manuscript and/or supporting information.
