# Supplementary material for: Bioinspired stability enhancement in deuterium-substituted organic–inorganic hybrid perovskite solar cells
Source: PNAS Nexus. 2023 May 16;2(5):pgad160. doi: 10.1093/pnasnexus/pgad160 (PMC10226519; doi:10.1093/pnasnexus/pgad160)
Supplement: pgad160_Supplementary_Data [file pgad160_supplementary_data.pdf]

Supplemental Materials

Bioinspired stability enhancement in deuterium-substituted organic-inorganic hybrid perovskite solar cells

Jinhui Tong,[1†] Xun Li,[2†] Jianxin Wang,[2] Haiying He,[3]* Tao Xu,[2*] Kai Zhu[1*]

[1]Chemistry and Nanoscience Center, National Renewable Energy Laboratory, Golden, Colorado 80401, USA

[2]Department of Chemistry and Biochemistry, Northern Illinois University, DeKalb, Illinois 60115, USA

[3]Department of Physics and Astronomy, Valparaiso University, Valparaiso, Indiana 46383, USA

[*]Corresponding authors: Kai.Zhu@nrel.gov (K.Z.); txu@niu.edu (T.X.); haiying.he@valpo.edu (H.H.)

[†]These authors contributed equally to this work.

Note 1: The available source of deuterium (D) on the Earth and a technoecono analysis (TEA) for using deuterium-substituted A-site cations in perovskite solar cells at scale:

There are one D out of 6700 H atoms in ocean water, and the total amount of D in the ocean is 2.4×10^{19} mol. Meeting the power production of 1 GW by using the typical $FAPbI_3$-based PSCs (550-nm-thick active layer) with an average PCE of 20% would require the amount of D from ~7×10^{4} mol (714 kg) of D_2O. This suggests that the needed amount of D for perovskite solar cells is negligible even at the TW level compared with the total amount of D in the ocean. Note that annual world D_2O production is about 1000 tons due to limited demand.[1] The cost of D_2O will be around $570K per 1GW perovskite solar cells, even using the high price of D_2O for scientific lab use, $797/kg at Apolloscientific (https://store.apolloscientific.co.uk/product/deuterium-oxide-999-atom-d-1l-bottle accessed on 1/05/2023). In contrast, the materials cost for 1GW perovskite solar cells (assuming 20% PCE) using regular hydrogen-based A-site cations is estimated to be $400M.[2] Thus, the replacement of H with D for an extra $570K/GW is negligible investment in materials compared to the extended lifetime it can provide. The actual cost to extract D_2O can be reduced to as low as $300/kg.[3] Also noted is that the replacement of the active H on nitrogen atoms in formamidilium (FA), a major A-site cation in perovskite solar cells, can be readily achieved by soaking the FAI in D_2O followed by recrystallization. The deuterium concentration in the remaining mother liquid can be readily recycled and re-enriched by the existing industrial methods and infrastructures for D_2O production for large-scale manufacturing. In this case, the efficiency of D_2O usage can be maximized.

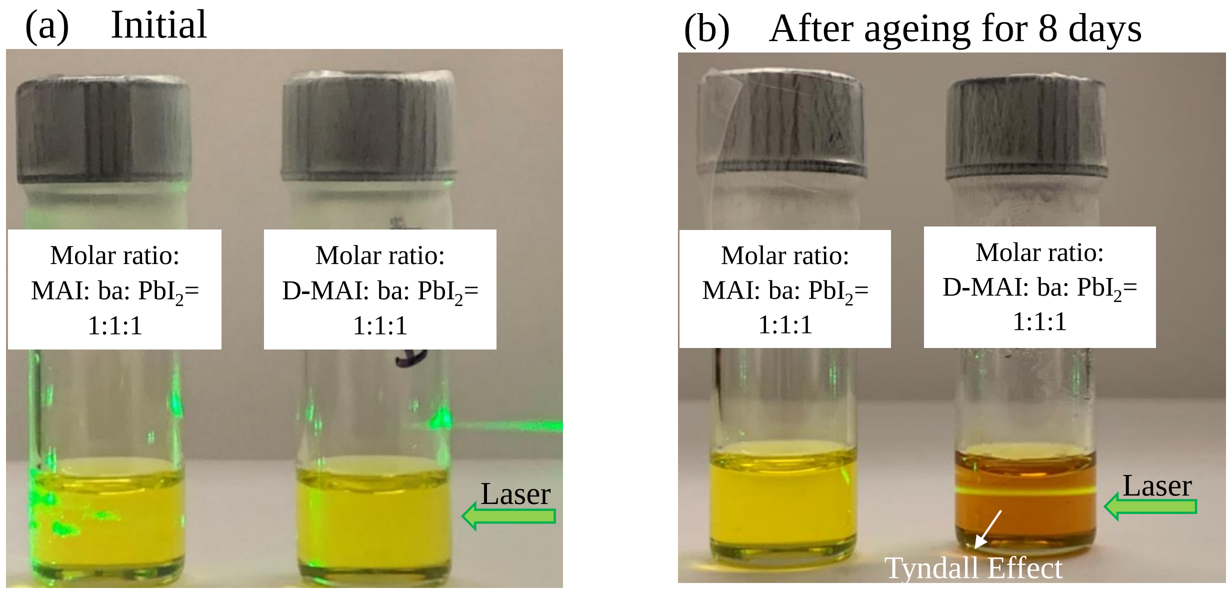

Fig. S1. (a) Photograph of the initial perovskite precursor solutions, which were light yellow and completely clear. (b) After aging for 8 days, precipitates formed in the D-$MAPbI_3$ precursor solution, clearly visualized by the Tyndall effect.

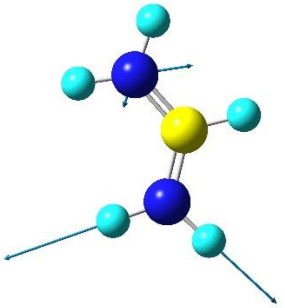	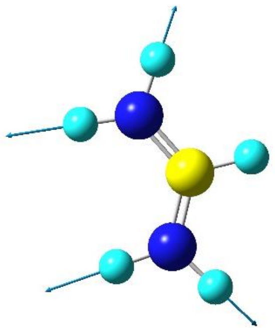
ν_1	ν_2
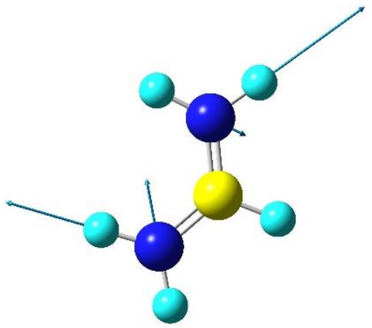	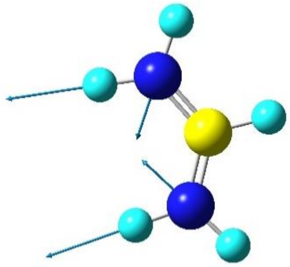
ν_3	ν_4

Fig. S2. Illustration of vibrational modes related to N-H bond stretching in FA. Symbols for atoms: C in yellow, N in navy blue, and H in light blue.

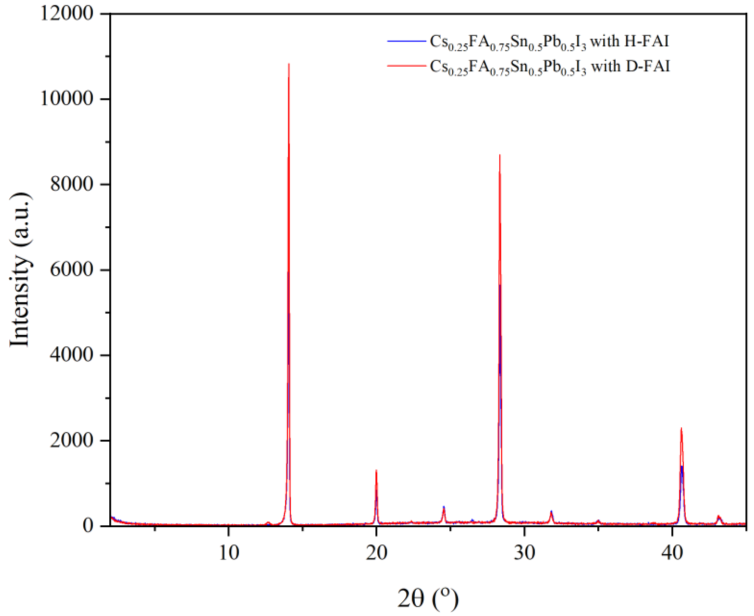

Fig. S3. XRD of H-FAI- and D-FAI-based narrow-bandgap $Cs_{0.25}FA_{0.75}Sn_{0.5}Pb_{0.5}I_3$ perovskite.

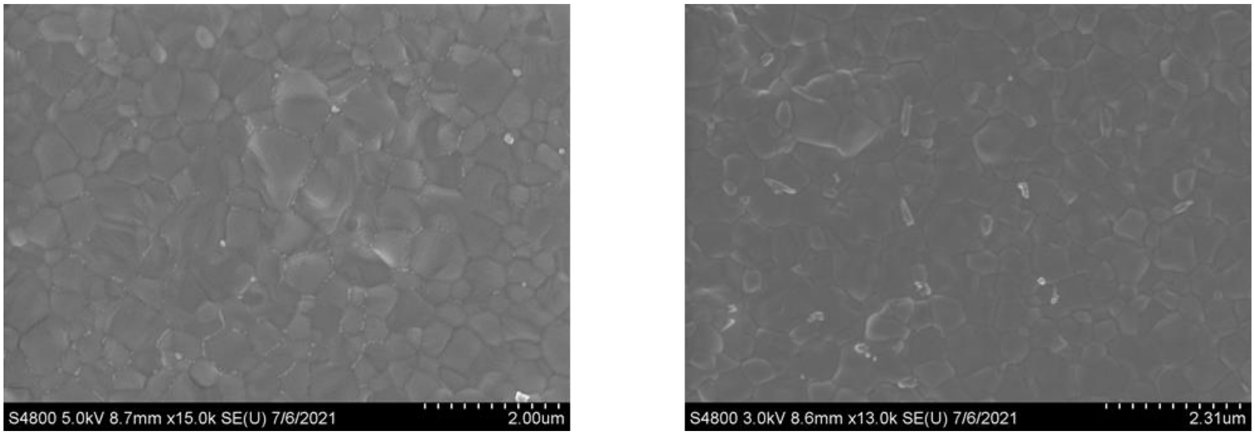

Fig. S4. SEM of H-based (left) and D-based (right) narrow-bandgap $Cs_{0.25}FA_{0.75}Sn_{0.5}Pb_{0.5}I_3$ perovskite.

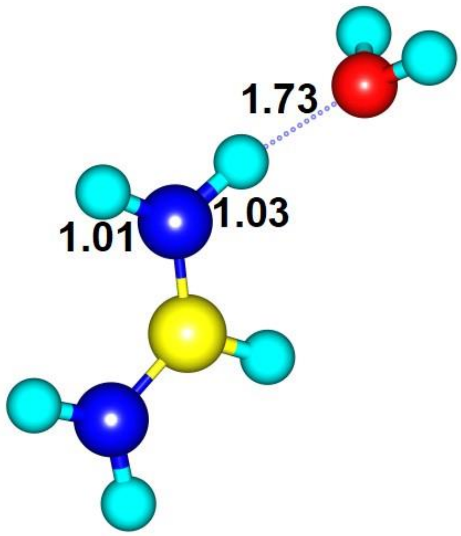

Fig. S5. Optimized structure of the FA···H_2O complex. Symbols for atoms: C in yellow, N in navy blue, H in light blue, and O in red.

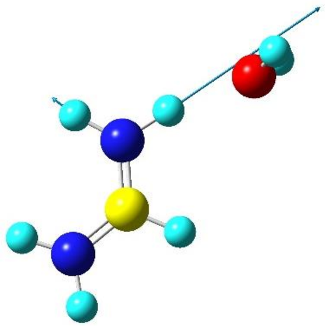	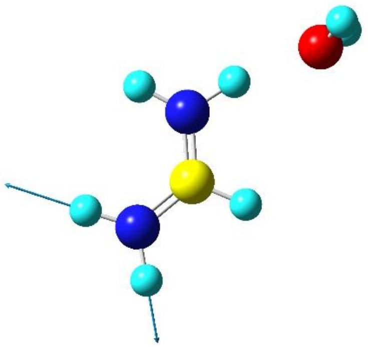
ν_1	ν_2
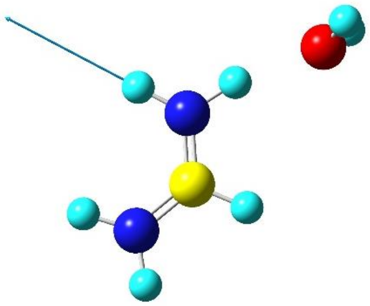	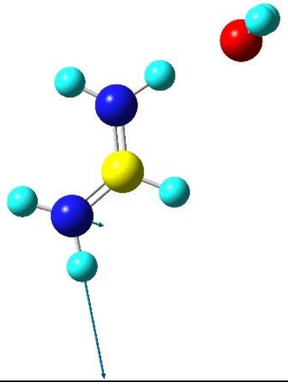
ν_3	ν_4

Fig. S6. Illustration of vibrational modes related to N-H bond stretching in the FA···H_2O complex. Symbols for atoms: C in yellow, N in navy blue, H in light blue, and O in red.

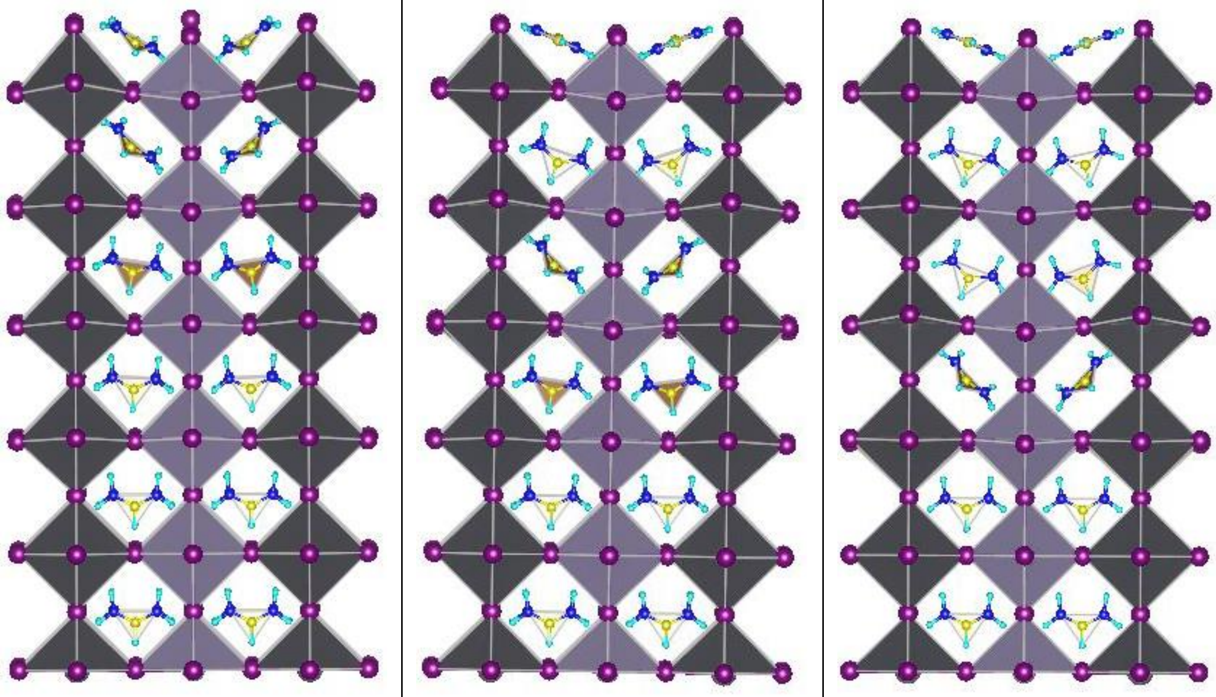

V_{Sn}^{2-} at 1st layer	V_{Sn}^{2-} at 2nd layer	V_{Sn}^{2-} at 3rd layer

Fig. S7. Structures of the defective systems with Sn^{2+} vacancies (V_{Sn}^{2-}) at the first, second, and third layers of the $FASn_{0.5}Pb_{0.5}I_3$ (001) surface. Symbols for atoms: C in yellow, N in navy blue, H in light blue, I in purple, Pb in gray, and Sn in light pink.

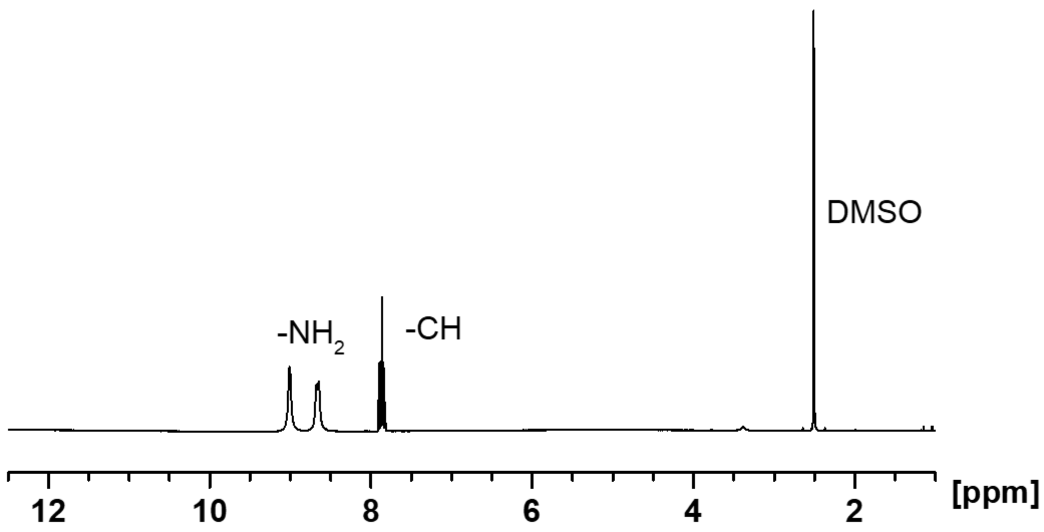

Fig. S8. $CH(NH_2)_2I$ was dissolved in d6-DMSO, and the peak at 2.5096 ppm corresponds to the signal of the inevitable residual undeuterated DMSO in the d6-DMSO solvent. The peaks at 9.0093 ppm and 8.6487 ppm correspond to hydrogen in $–NH_2$ of $CH(NH_2)_2I$. The peak at 7.8632 ppm corresponds to hydrogen in –CH of the $CH(NH_2)_2I$ compound. The ratio of the integrated peak areas for H-C and H-N is approximately 1:4, in good agreement with the molecular structure of FAI.

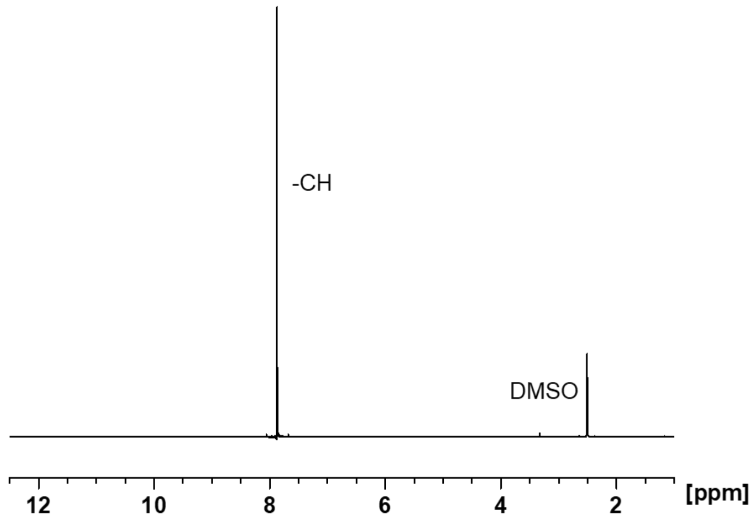

Fig. S9. $CH(ND_2)_2I$ was dissolved in d6-DMSO, and the peak at 2.5096 ppm corresponds to the signal of the inevitable residual undeuterated DMSO in the d6-DMSO solvent. The peak at 7.8716 ppm corresponds to hydrogen in –CH in $CH(ND_2)_2I$. The absence of peaks in the 8.4–9.2 ppm range indicates the fully deuterated H-N in $CH(ND_2)_2I$.

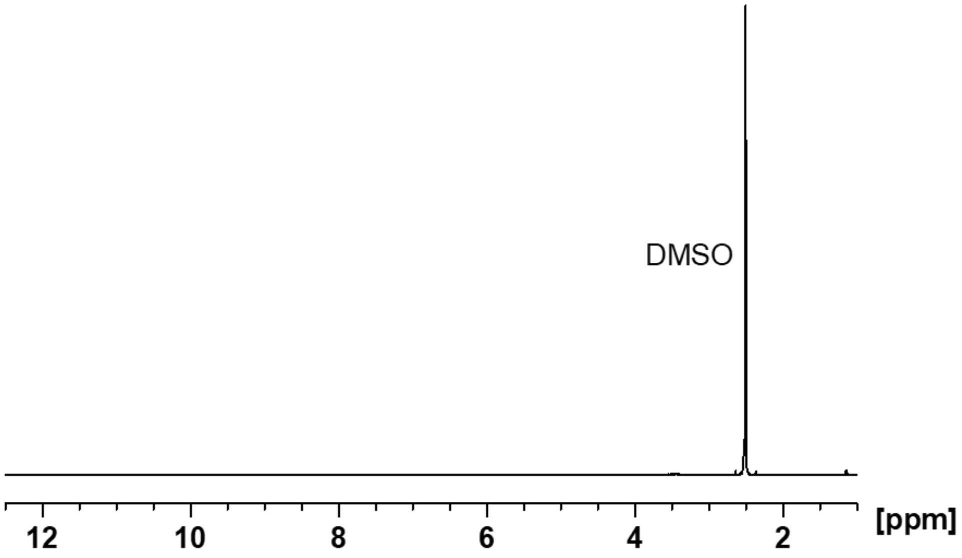

Fig. S10. ^{1}H NMR spectrum of the pristine DMSO-D6 solvent without any samples dissolved. The peak at 2.5096 ppm is the signal for the residual d5-DMSO in commercial d6-DMSO solvent. This control experiment suggests that the solvent does not have any water peak or any other peaks.

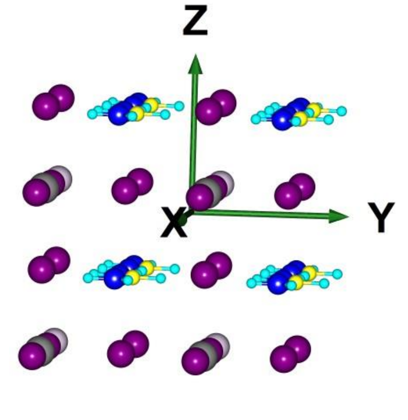	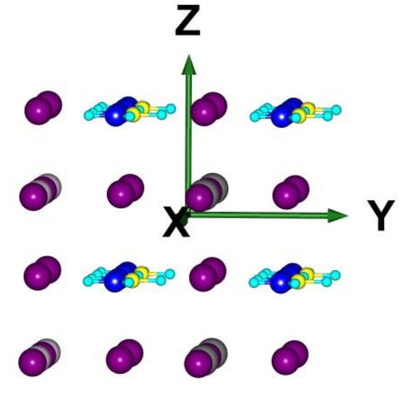	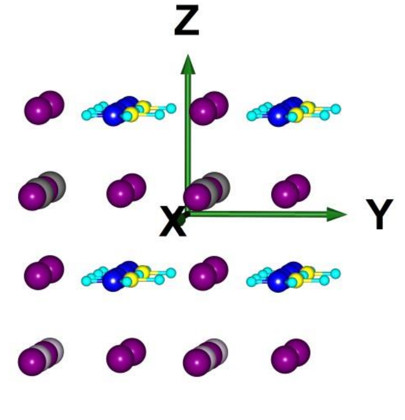
E = 0 eV	E = 0.0002 eV	E = 0.0003 eV

Fig. S11. $FASn_{0.5}Pb_{0.5}I_3$ bulk with different Sn ordering. The relative energy difference per formula unit is listed. Symbols for atoms: C in yellow, N in navy blue, H in light blue, I in purple, Pb in gray, and Sn in light pink.

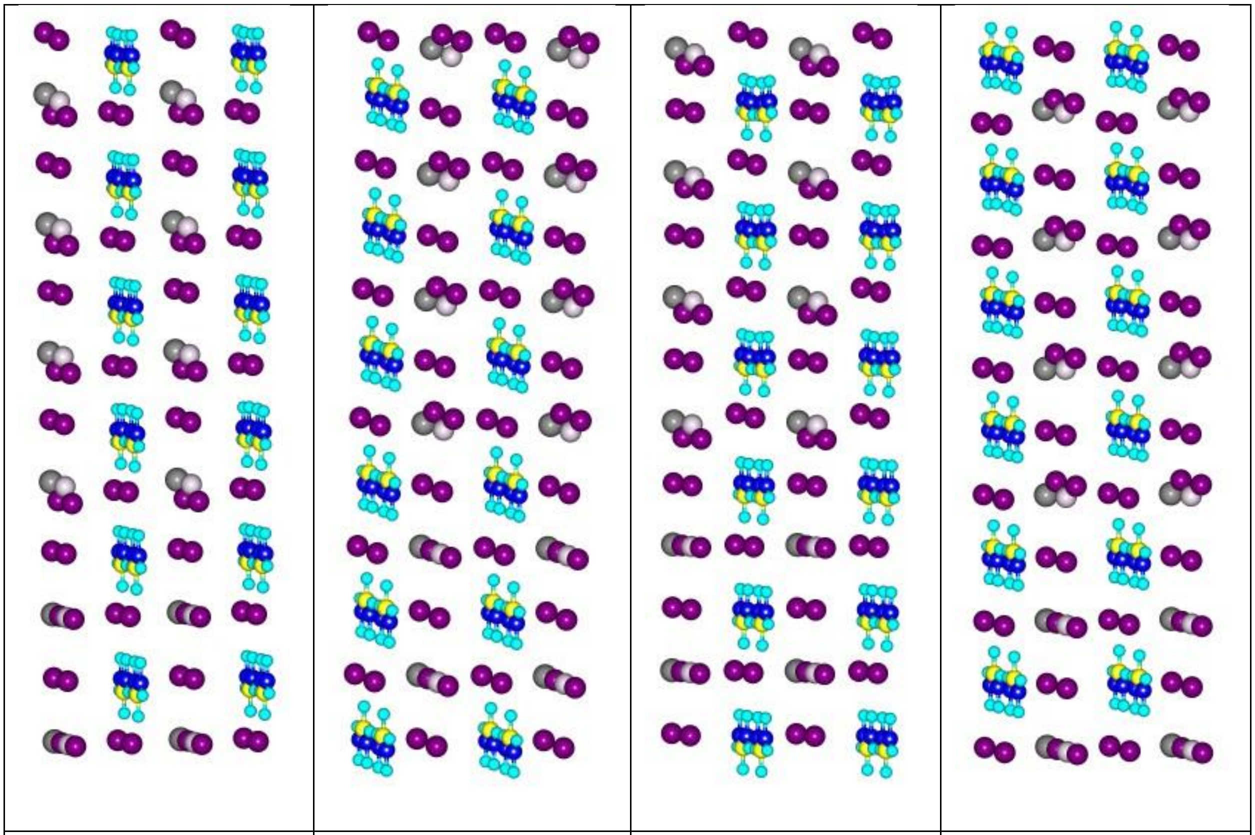

(a) E = 0 eV | (b) E = 0.30 eV | (c) E = 0.82 eV | (d) E = 1.40 eV

Fig. S12. Illustration of $FASn_{0.5}Pb_{0.5}I_3$ surfaces and associated surface energies for (a) (001) surface with FAI termination, (b) $(00\bar{1})$ surface with PbI_2 termination, (c) (001) surface with PbI_2 termination, and (d) $(00\bar{1})$ surface with FAI termination. The relative energy difference is listed. Symbols for atoms: C in yellow, N in navy blue, H in light blue, I in purple, Pb in gray, and Sn in light pink.

Table S1. Calculated vibrational frequencies (cm^{-1}) for H-based and D-based FA performed at the B3LYP/6-311+G(3df,2p) level with the van der Waals dispersion interaction included. A scaling factor of 0.9670 is applied to the frequencies.

	**N-H**	**N-D**	**Ratio of ν(N-D)/ν(N-H)**
ν_1	3415	2471	0.723
ν_2	3429	2486	0.725
ν_3	3536	2619	0.741
ν_4	3537	2624	0.742

Table S2. Calculated vibrational frequencies (cm^{-1}) for H-based and D-based FA···H_2O performed at the B3LYP/6-311+G(3df,2p) level with the van der Waals dispersion interaction included. A scaling factor of 0.9670 is applied to the frequencies.

	**N-H**	**N-D**	**Ratio of ν(N-D)/ν(N-H)**
ν_1	3053	2240	0.733
ν_2	3434	2486	0.724
ν_3	3476	2558	0.736
ν_4	3551	2632	0.741

Table S3. Itemized corrections to the Gibbs free energy (ΔG_{corr}) of the defect formation energy of V_{Sn}^{2-} on $FASn_{0.5}Pb_{0.5}I_3$ (001) surfaces (labelled as H-FAI and D-FAI for H-based and D-based, respectively) at T = 298.15 K. Frequency calculations are performed using the periodic PBE-D3 method as implemented in VASP for the pristine (001) surface with FA-I termination and the defective surface with V_{Sn}^{2-} at the top layer .

	ZPE (eV)	*C*v (0→T) (eV)	*S* (eV/K)	-*T*S* (eV)	ΔG_{corr} (eV)
**H-FAI: pristine**	30.959	2.051	0.0131	-3.919	29.091
**H-FAI: with V_{Sn}^{2-}**	31.085	1.997	0.0126	-3.756	29.326
**D-FAI: pristine**	24.827	2.538	0.0179	-5.348	22.017
**D-FAI: with V_{Sn}^{2-}**	25.124	2.333	0.0149	-4.448	23.009

References in Supporting Materials

1. M. Lozada-Hidalgo *et al.*, Scalable and efficient separation of hydrogen isotopes using graphene-based electrochemical pumping. *Nature Communications* **2017**, *8,* 15215.

2. Čulík, P.; Brooks, K.; Momblona, C.; Adams, M.; Kinge, S.; Maréchal, F.; Dyson, P. J.; Nazeeruddin, M. K. Design and Cost Analysis of 100 MW Perovskite Solar Panel Manufacturing Process in Different Locations, *ACS Energy Lett.* **2022**, *7*, 3039−3044.

3. Miller, A. I. Heavy water: a manufacturers' guide for the hydrogen century. *Canadian Nuclear Society Bulletin* **2001**, *22,* 1-14.
